# A three-dimensional printed porous implant combined with bone grafting following curettage of a subchondral giant cell tumour of the proximal tibia: a case report

**DOI:** 10.1186/s12893-019-0491-y

**Published:** 2019-02-28

**Authors:** Minxun Lu, Jie Wang, Fan Tang, Li Min, Yong Zhou, Wenli Zhang, Chongqi Tu

**Affiliations:** Department of Orthopedics, West China Hospital, Sichuan University, No. 37 Guoxue Street, Chengdu, 610041 People’s Republic of China

**Keywords:** Proximal tibia, Giant cell tumour, Epiphysis, Subchondral bone, Three-dimensional printed implant, Porous, Bone graft, Case report

## Abstract

**Background:**

Subchondral bone is commonly affected in cases of giant cell tumour (GCT) of the proximal tibia. Numerous studies have stated that retaining a sufficient subchondral bone layer could decrease the possibilities of postoperative degenerative changes and mechanical failure of the knee joint. However, the most commonly used methods of cement packing only or cement packing combined bone grafting have some limitations regarding the protection of subchondral bone after surgery. Our paper describes our attempt to reconstruct a tumorous defect associated with the subchondral area in the proximal tibia with a three-dimensional (3D)-printed porous implant combined with bone grafting and our evaluation of the short-term outcomes.

**Case presentation:**

A 42-year-old man with a Campanacci grade II GCT visited our institution for initial assessment. Radiographs showed a tumour located at the epiphyseal part of the proximal tibia, and had invaded the subchondral area. Considering that the residual subchondral bone had to be protected, we used an autograft to ensure a high integration rate between the graft and host subchondral bone. We also used three-dimensional (3D) printing technology to design and fabricate a personalized porous implant to mechanically support the graft and subchondral area and help avoid degenerative changes and mechanical failure. At the last follow-up at 29 months postoperatively, the patient had satisfactory limb function and no further damage was seen to the subchondral area and articular surface.

**Conclusions:**

The 3D-printed porous implant combined bone grafting may be a feasible option in the reconstruction of defects that are close to the subchondral area following extensive intracurettage of GCTs. Moreover, it can result in good postoperative function and low complication rates. However, consistent follow-up is required to clarify its long-term outcomes.

## Background

Giant cell tumour (GCT) of the bone is a benign but locally aggressive tumour with a relatively high recurrence rate after primary treatment [1]. GCTs mainly occur between the age of 20 and 40 years [2]. Epiphyseal regions of the long bones are the most commonly affected, especially the distal femur and proximal tibia [3]. Furthermore, subchondral bone around the knee is commonly invaded and/or destroyed by Campanacci Grade I and II GCTs of the epiphyseal region. Previous studies have stated that a lower quantity of subchondral bone remaining can result in postoperative mechanical failure, including deformity, fracture, and even collapse of the articular surface [4], which may result in discomfort and poor joint function of the knee [4, 5]. Therefore, protecting the retained subchondral bone after extensive intracurettage and postoperatively promoting the ingrowth of subchondral bone are the most crucial principles for the treatment of GCTs with Campanacci Grade I and II that occur around the knee. Currently, cement packing [6], bone grafting [7], and a combination of cement packing and bone grafting [5] are the most common and acceptable choices for reconstruction. Cement packing was initially the most popular method, the lack of bone inducibility and conductibility is the major disadvantage of the method. In contrast, bone grafting provides excellent biocompatibility and enables biological reconstruction. However, mechanical strength is often lacking in bone grafts [8]. Therefore, combined bone grafting and cement packing seems to be the best option. Subchondral bone is protected from cement deconstruction by the bone graft that is placed between the subchondral bone and cement [9], but the problems related to the graft-cement interface still exist in this combination. We proposed the use of a three-dimensional (3D)-printed porous implant with scaffold structure combined with bone grafting, which would ideally enable a high level of osseointegration, therefore resulting in an improved biological interface between the graft and implant after reconstruction when compared to cement packing combined bone grafting. To the best of our knowledge, no studies have been performed on this subject. Our paper describes a case wherein reconstruction was performed with a combined bone grafting and 3D-printed porous implant for a tumour-induced defect of the proximal tibia in a patient with Campanacci grade II GCT.

## Case presentation

### History

A 42-year-old man complained of progressive pain in the left knee region without any limitation in daily activity for 3 months. He visited our institution for initial assessment and underwent radiographic investigation in January 2016. A large radiolucent area caused by osteolytic deconstruction in the epiphyseal part of the left proximal tibia was observed. Computed tomography (CT), magnetic resonance imaging, Tomosynthesis-Shimadzu metal artefact reduction technology (T-SMART), bone scan, and biopsy were then performed to assess the precise diagnosis, safe border of the lesion, condition of the surrounding bone, and lung condition. The lesion was finally diagnosed as Campanacci Grade II GCT with no pulmonary metastasis. No fracture of the subchondral bone was detected on the CT scan.

Before surgery, the pain, knee joint function, percentage of affected area of the subchondral bone, and thickness of the residual subchondral bone layer were precisely evaluated. The pain at rest was evaluated according to the Visual Analog Scale (VAS) in which 0 represents no pain and 10 represents the worst pain imaginable. The range of knee joint motion was also recorded. The percentage of affected area of the subchondral bone was calculated as described by Chen [10]. The shortest distance from the articular surface to the nearest margin of the tumour on radiographs was defined as the thickness of the residual pre-operative subchondral bone layer. This distance was measured with imaging generated using the 3D-CT scan and Tomosynthesis-Shimadzu metal artefact reduction technology (T-SMART). Considering the poor condition of the retained subchondral bone, implantation using a 3D-printed scaffold structure and supplemental bone graft were performed to prevent further damage on the subchondral bone that could potentially be caused by the friction between the cement and subchondral bone when cement packing and to avoid formation of non-biological and rigid graft-cement interface that occurs after cement packing combined bone grafting. This study was approved and monitored by the local Ethical Committee. The patient was informed about the risks and benefits of the treatment, after which he provided written informed consent.

### Implant design and fabrication

**Fig. 1:**
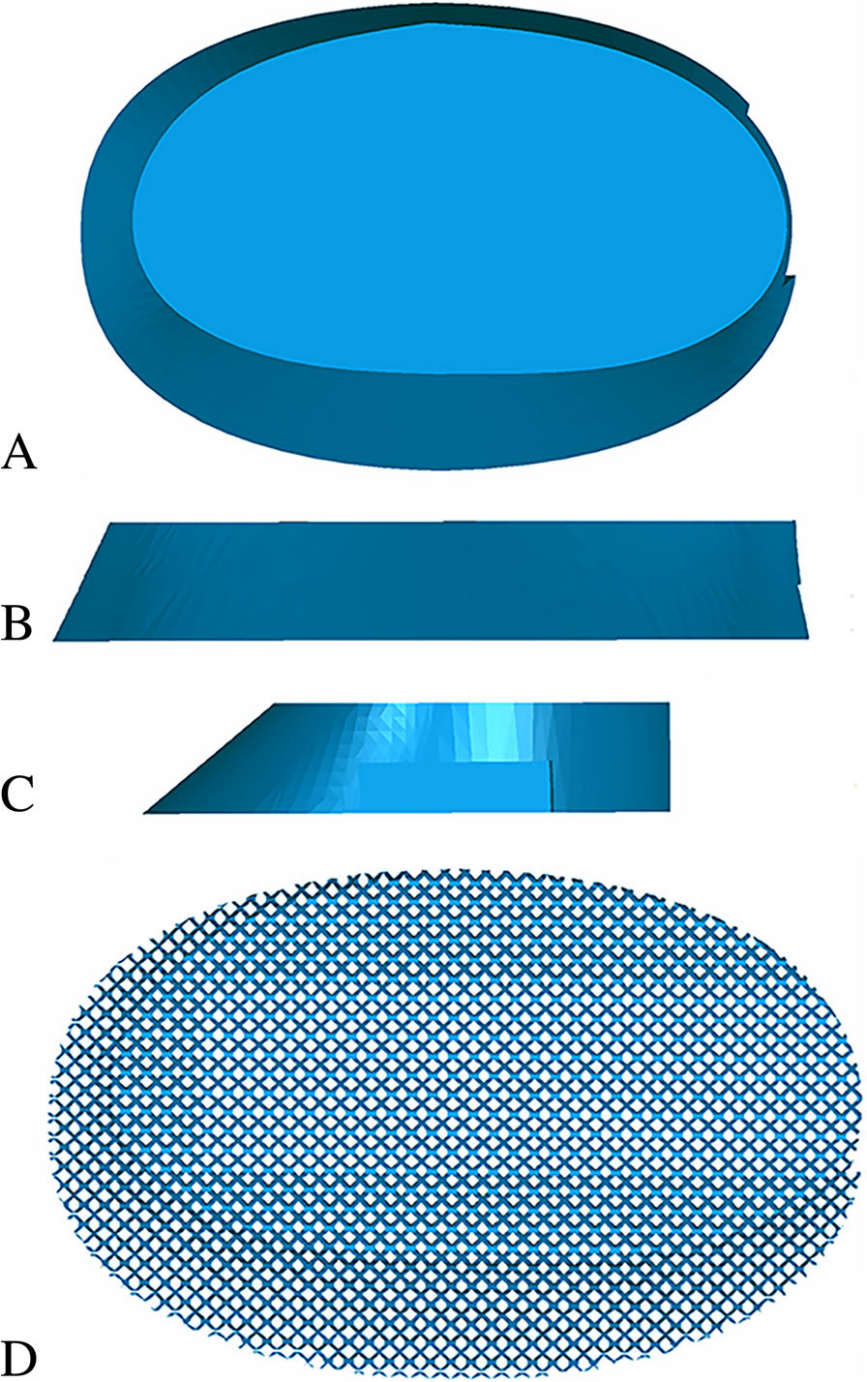
**a**-**d**. 3D model of bearing plate: (**a**) top-bottom view; (**b**) anterior-posterior view; (**c**) lateral view; (**d**) plate with scaffold structure

**Fig. 2:**
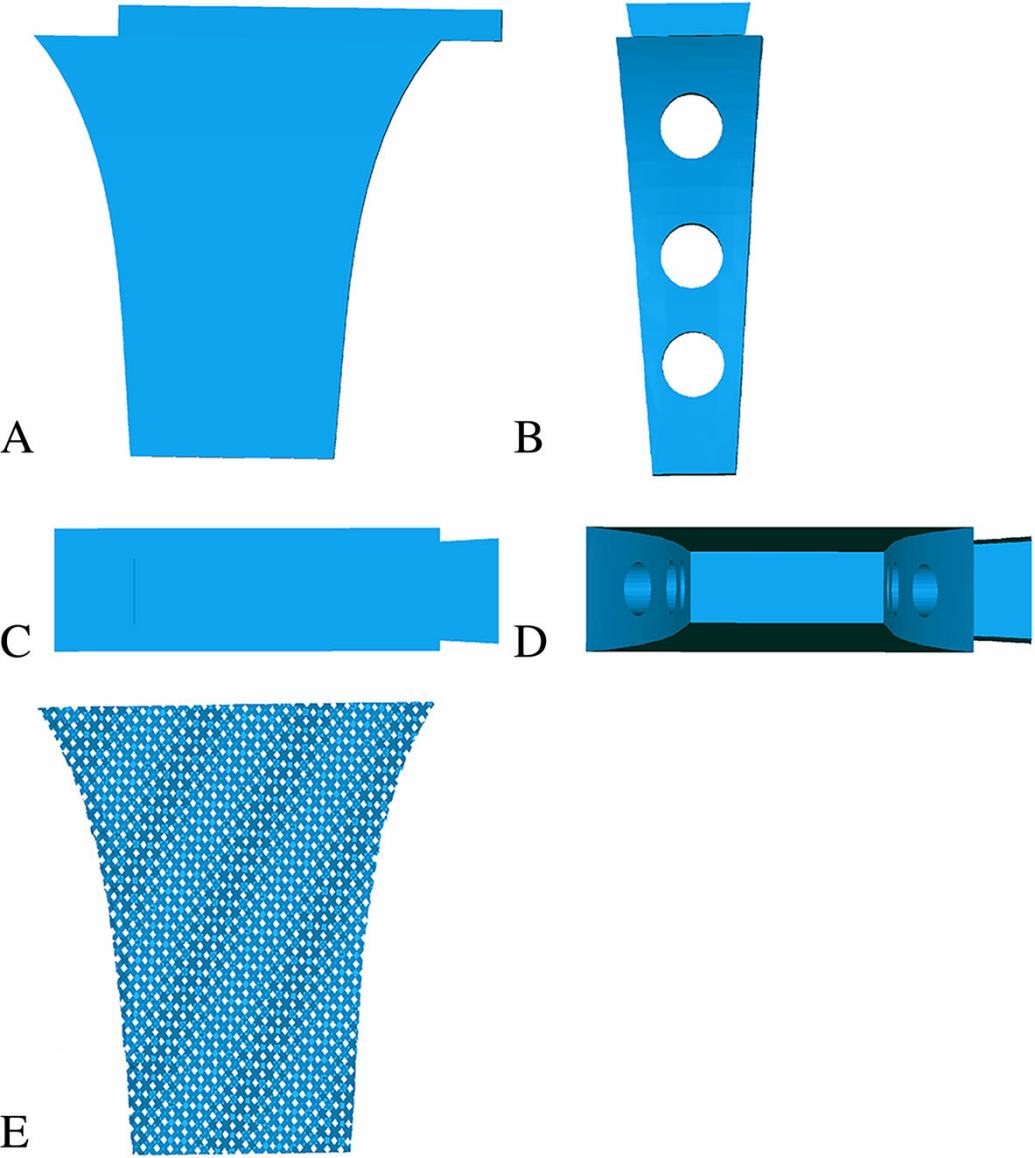
**a**-**e**. 3D model of strut: (**a**) anterior-posterior view; (**b**) lateral view; (**c**) top-bottom view; (**d**) bottom-top view; (**e**) strut with scaffold structure

**Fig. 3:**
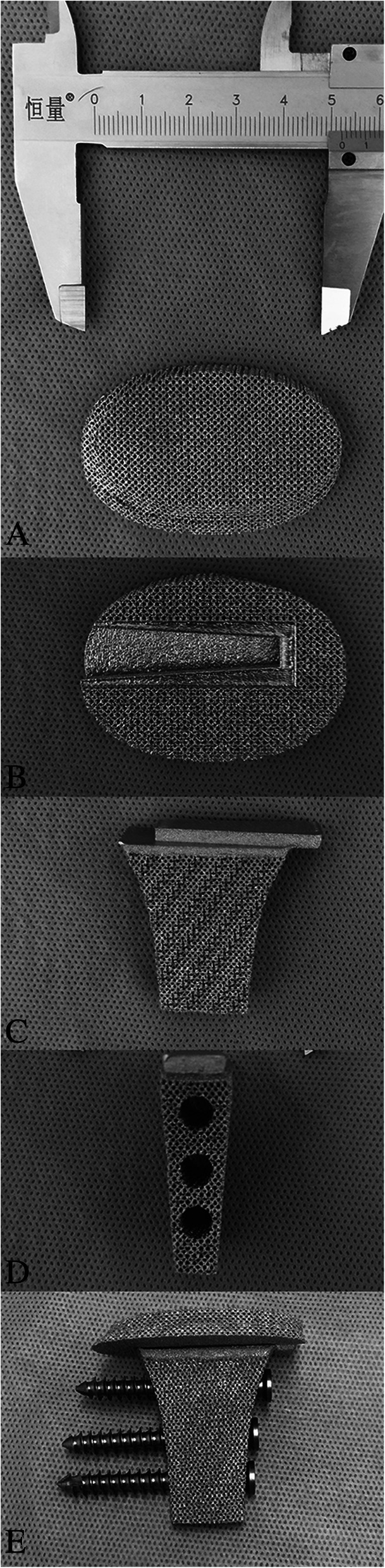
**a**-**e**. Components of implant: (**a**) top-bottom view of plate; (**b**) bottom-top view of plate; (**c**) anterior-posterior view of strut; (**d**) lateral view of strut; (**e**) Assembled implant with screws

The implant was fabricated by Chunli Co, Ltd., Tongzhou, Beijing, China. The primary aims when designing the implant were: maintaining the articular surface in the proper position, preventing collapse of the subchondral bone and joint surface, and promoting healing between the subchondral bone and graft. Therefore, the implant needed a porous bearing surface with a great ability of osseointegration and a porous strut providing effective axial support as per the appropriate length. To analyse and evaluate the size and shape of cavity caused by tumorous deconstruction, importing 3D CT scan data into the Mimics V17.0 software (Materialise Corp. Belgium) was initially performed. The possible size and shape of the implant was preliminarily calculated with the Mimics software using the sagittal, coronal, and transverse images obtained via the CT scan. The implant prototype using the initial data was created by Solidworks 2016 (Dassault Systemes, France). To ensure satisfactory fitting in the tumorous cavity of the proximal tibia, the size and shape of the implant was optimized via computer simulation of implantation. Considering the convenience of implantation, the final implant consisted of two parts that could be assembled, including a truncated ellipsoid cone-shaped plate with 10 mm thickness (Fig. 1), and a square frustum-shaped strut with 45 mm axial length (Fig. 2). To assemble the two parts tightly, a specialized slideway, which can mechanically prevent dropping and slipping of the strut, was created on the bearing plate; the appropriate slider was required to be located on the strut. Three screw holes were designed to thoroughly affix the strut to the unaffected cortex of the proximal tibia. Except for the specialized slideway and slider, the whole implant was designed using a scaffold structure. For fabricating the implant, Electron Beam Melting technology (ARCAM Q10, Sweden) was applied (Fig. 3).

### Surgery

**Fig. 4:**
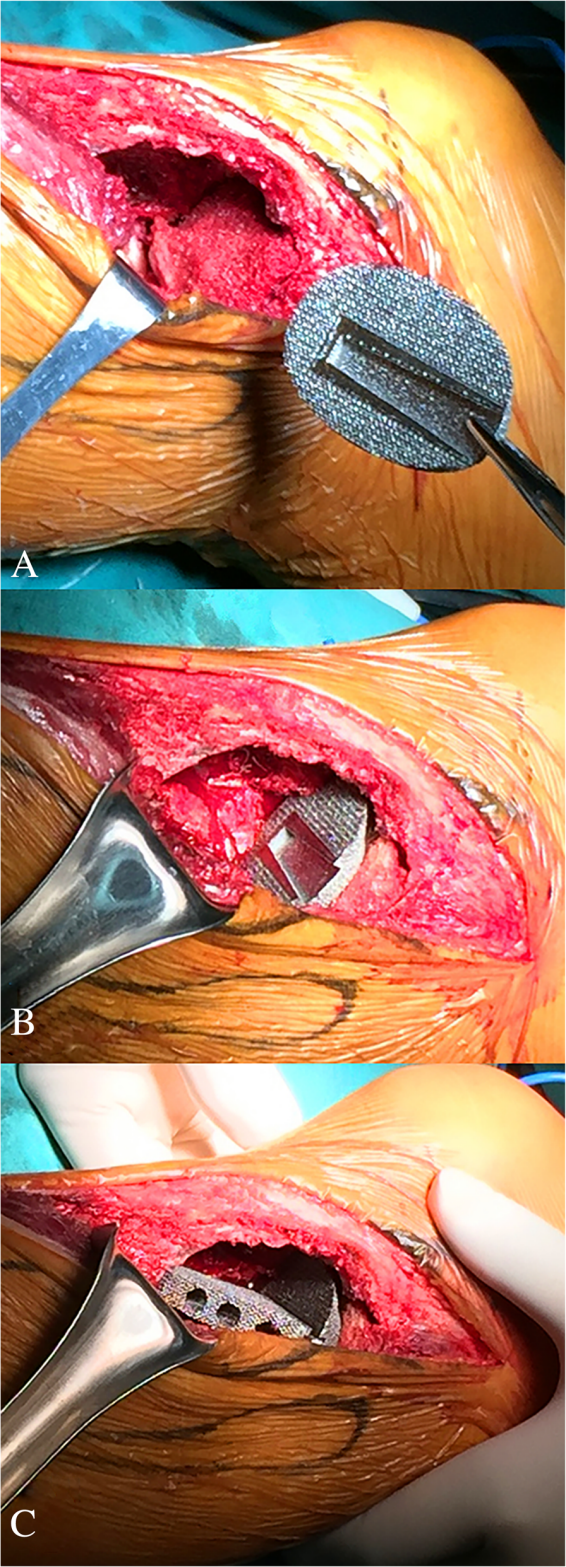
**a**-**c**. Intraoperative pictures: (**a**) indicating graft transplantation ; (**b**) indicating implantation of plate; (**c**) indicating implantation of strut

**Fig. 5:**
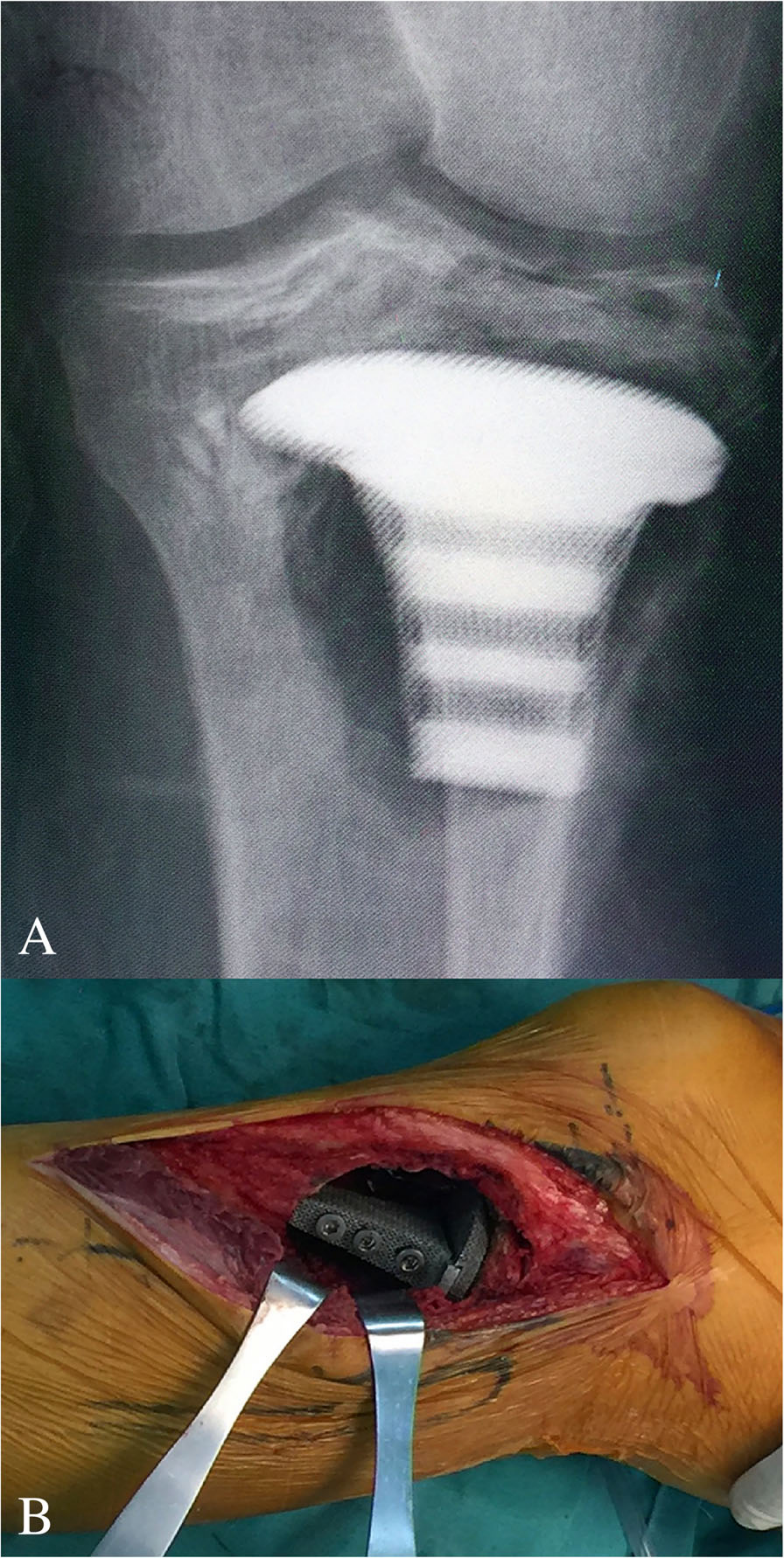
**a**-**b**. Intraoperative pictures: (**a**) confirming the location of implant by the X-ray; (**b**) indicating screws fixation after the confirmation of implant position

The surgery was performed by a senior surgeon (Chongqi Tu). With the patient under general anaesthesia, the GCT of proximal tibia was exposed through a lateral approach. Before intracurettage, mini drill bites were used to minimize the bone loss caused by the drill. To reduce the rate of local recurrence, the invaded soft tissue over the tumour cortex was removed. Then, extensive intracurettage was performed with a high-speed burr and electrocauterization repeatedly to completely remove the tumour. After the curettage was completed, phenol, hydrogen peroxide solution, and distilled water pulse irrigation was used to rinse the cavity. The bone graft obtained from the patient’s iliac bone was placed directly under the residual subchondral bone to provide biomechanical support. Then, a bearing plate with a porous surface was implanted into the cavity just under the graft and the strut was inserted into the cavity through the slideway on the porous plate (Fig. 4). After confirming both locations of the plate and strut of the implant, the three screws were fixed to the contralateral side of the proximal tibia cortex to enhance the primary stability of the implant (Fig. 5).

### Postoperative management

Considering insufficient primary stability in the early postoperative period, the patient was allowed non-weight-bearing standing and walking with two crutches 3 weeks after surgery. However, range of motion exercises of the knee were performed from postoperative week 4. Partial weight-bearing with crutches was encouraged from 6 weeks postoperatively, followed by gradual full weight-bearing. The patient was followed up every month for the first 3 months, then every 3 months thereafter. The postoperative pain, range of motion, degenerative changes, thickness of subchondral bone layer, oncology outcomes, and radiographs were assessed at each follow-up visit to verify the outcomes of the 3D-printed implant reconstruction.

### Outcome

**Fig. 6:**
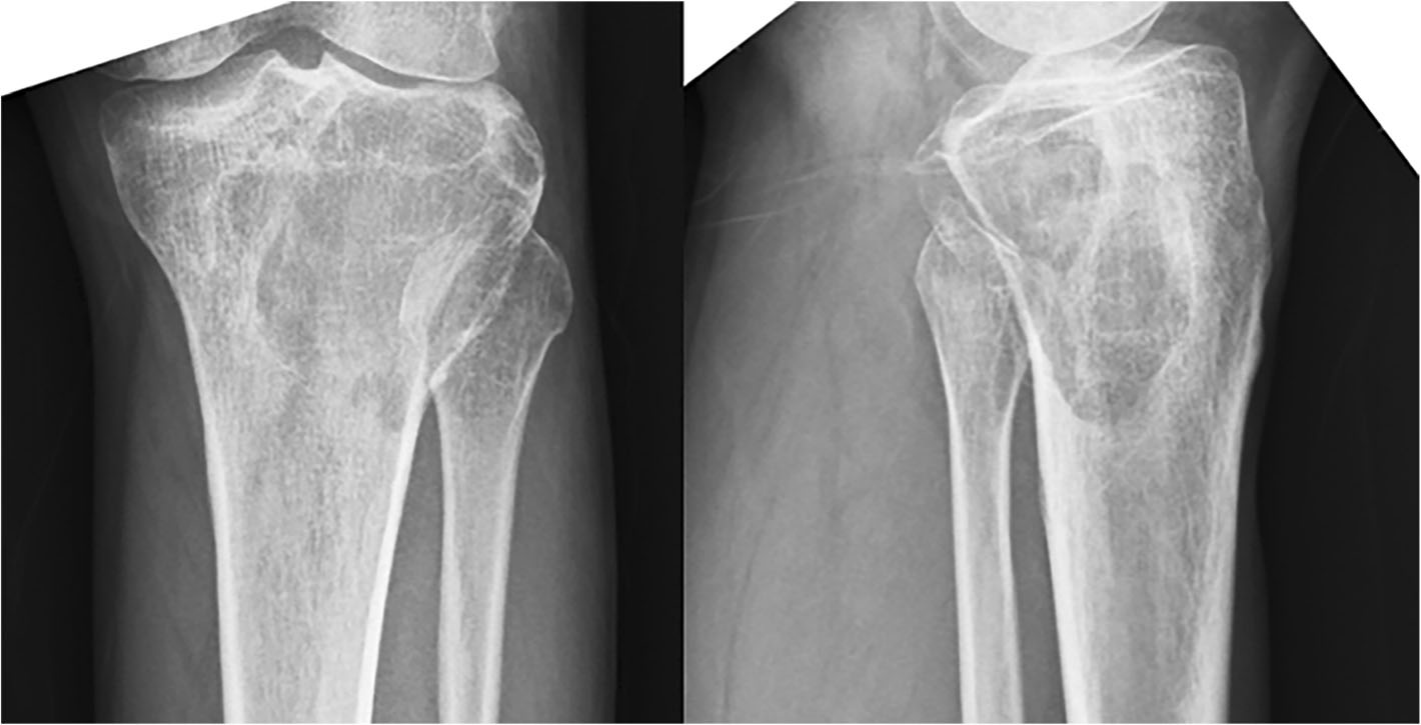
A-B. Method used to measure the area of the subchondral bone affected by the giant cell tumor of the bone: (A) The width of epicondyle to middle of joint (A) and the width of affected subchondral area (a) were measured in the anteroposterior plane; (B) The whole width of joint (B) and the width of affected subchondral area (b) were measured in the lateral plane; (a × b/A × B) × 100 =% of the subchondral bone area affected by tumor

**Fig. 7:**
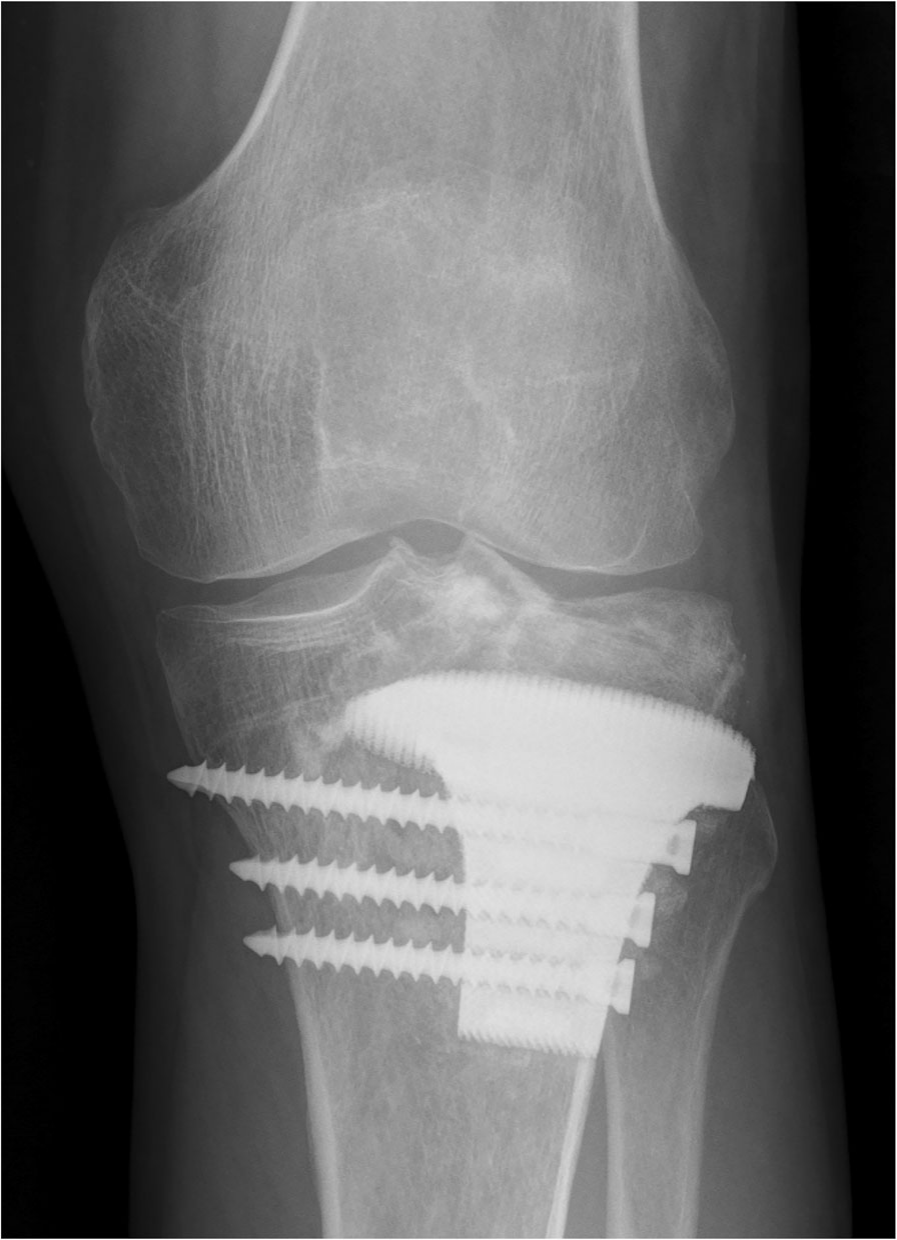
Anteroposterior radiograph of left tibia 29 months after 3D-printed implant reconstruction

The patient was followed up over a duration of 29 months. The preoperative VAS score was 7, which decreased to 0 after the surgery. There was no detectable difference in the range of knee motion, which was satisfactory in the preoperative and postoperative period, with a normal range. Before the surgery, the percentage of the affected subchondral area was 48.2% (Fig. 6) and the thickness of the subchondral bone layer was less than 3 mm. However, because of the excellent integration between the graft and retained subchondral bone, the subchondral bone layer was noticeably thicker at the last follow-up, which is 10.9 mm. Furthermore, no degenerative changes were observed during the follow-up period. No fracture or collapse of articular surface was detected. No local recurrence or lung metastasis occurred. Radiography indicated that the implant fitted well with the tibia and no signs of complications, including breakage and displacement, were found (Fig. 7).

## Discussion and conclusion

Campanacci grade I or II GCTs have a lower recurrence rate and are less aggressive than grade III GCTs. However, the subchondral bone or even articular surface might be affected when grade I or II GCTs are located at the epiphyseal part of the proximal tibia. Several studies have indicated that insufficient postoperative subchondral bone thickness would increase the risk for degenerative changes, deformity, fracture, and even collapse of the knee joint [11–13]. According to a study by Abdelrahman [4], the probability of degeneration in the group where the thickness was less than 10 mm was 2.5 times that of the group where the thickness was more than 10 mm. The most acceptable explanation related to the interaction between mechanical failure and thickness of the subchondral bone layer might be that the thinner subchondral layer has a lower shock-absorbing capacity [7, 14–16]. Considering the ages of the patients with GCTs, maintaining a normal articular surface and preventing further damage to the subchondral bone are very crucial. Although extensive intracurettage might be a potential risk factor for operative damage on the subchondral bone, this procedure was necessary to maintain a lower recurrence rate after treatment of GCTs. However, the best reconstruction option was still controversial, especially regarding the aspect of subchondral bone and articular surface protection. Three methods for filling the cavity have been reported and discussed [4–7, 17, 18]. Among these, cement packing is the most frequently used method. However, cement has no ability to induce the growth of surrounding bone, and could instead result in the absorption of the surrounding bone because of the significant difference in the material properties between cement and bone. Consequently, a sclerotic rim would be inevitably apparent, separating the cement from the surrounding bone and subchondral bone layer [18]. Welch et al. [16] described that this rim could decrease the shock-absorbing capacity of the subchondral bone layer. Furthermore, the sclerotic rim could increase the micro-movement between the cement and bone, resulting in further damage to the surrounding bone such as subchondral bone. In contrast, an autograft or allograft is a better choice for protection of the subchondral bone. In a study by Benevenia et al. [19], nononcologic complications in patients of GCTs were clearly reduced after bone grafting in the subchondral bone area. However, the mechanical strength was a concern when packing alone. Therefore, degenerative changes and/or mechanical failure of the knee joint were also seen to occur with this approach. Furthermore, a higher rate of mechanical failure was seen when the cavity was filled using only bone graft when compared to when it was filled using cement packing only. In an animal model experiment [8], it was seen that the strength of the subchondral defect filled with cancellous bone was only slightly greater than that of the empty cavity. Therefore, the combination packing using graft and cement, which provides both mechanical strength and biological reconstruction, has been accepted by several researchers. It was seen that the sufficient support strength generated by cement packing and great bone conduction and induction provided by autograft/allograft would ensure a low incidence of postoperative degenerative changes and mechanical failure. This was also seen in the study by Teng et al. [5]. However, the complications related to non-biological bone-cement interface have still not been solved. We believe that 3D-printed porous implant reconstruction combined with bone graft would provide adequate support strength and excellent osseointegration and would therefore be a better method. In our case, we observed integration between the residual subchondral bone and graft, resulting in sufficient thickness of the postoperative subchondral bone layer to support the articular surface. This indicated a relatively low possibility of degenerative changes, fracture, or collapse of the knee joint in the future. Furthermore, no radiolucent area was detected in the graft-implant interface, indicating that the interface was well integrated. No degenerative changes or mechanical failure were seen during the follow-up period of 29 months.

In addition to the shape and thickness of the bearing plate, suitable strut length and appropriate settings with pore size and porosity to each part would be the main factors that enable good function with a low complication rate. The main principle of shape design of the bearing plate is enlarging the contact area between the plate and surrounding bone to provide strong primary stability and to induce bone growth more easily. When designing the bearing plate, osseointegration was the main priority. Thus, all settings related to scaffold structure were intended to increase and enhance the bone ingrowth by simulating the properties of trabecular bone. Torres-Sanchez [20] reported that a combination of pore size with a diameter of 500 μm and nearly 70% porosity would be a better option to simulate trabecular bone and to improve osseointegration. Therefore, the porous plate was designed with these parameters to provide an increased osseointegration ability. Regarding the plate, the location of the edges and the plate thickness are important factors. Although the edges of the plate inserted into the surrounding bone could generate supplemental primary stability, this edge design should be avoided in sites that have no cortex or insufficient cortical thickness. In our case, plate thickness was an important part of the design. It is obvious that the thinner plate would have weaker mechanical strength. However, a relatively thick plate with adequate support strength might be an obstacle for strut implantation, which would generate the major strength required to support the graft, subchondral bone, and articular surface. Therefore, we believe that a thickness of 10 mm would be a better choice.

Furthermore, some designing points of the strut were also crucial, including the length, screw hole, pore size, and porosity. Because the major function of the strut is to provide mechanical support, a suitable length should be precisely calculated and designed. If the strut is not long enough, the plate, graft, subchondral bone, and articular surface would collapse due to the lack of mechanical support. If the strut is longer than we needed, a greater amount of cortex will need to be removed to enlarge the potential window of implantation, which would cause meaningless bone loss. Compared to the plate, the settings with pore size and porosity of the strut were more likely to require focus on the mechanical strength to provide enough support. Therefore, the porous strut we used was designed with a pore size of 400 μm and 55% porosity, based on the design provided by Torres-Sanchez [20] to simulate the cortex.

Appropriate bone graft obtained from the patient’s iliac bone, placing the bearing plate with proper position, ensuring a stable connection between the plate and strut, and effective fixation of screws were the key procedures during the surgery. The removal of the graft cortex obtained from the iliac bone was necessary to achieve a well-integrated interface between the subchondral bone and graft. Furthermore, ensuring a proper shape of bone graft was also required to enlarge the contact area and to distribute the stress loaded on the subchondral bone more evenly. When placing the plate, we should ensure there is no gap between both interfaces, including subchondral bone-graft interface and graft-plate interface, because the bone ingrowth would be affected by the gap. After the plate was satisfactorily placed, the strut could be tightly assembled with the plate using a specialized slideway. Finally, we fixed the strut using three screws to the cortex of the proximal tibia to achieve increased primary stability.

We recognize the following limitations of this case report. The duration of follow-up is not yet sufficient to verify the long-term efficacy of this alternative design of implant and surgical techniques. More complications or problems might become apparent once these patients are followed up for a longer time. One case is insufficient to verify the advantages of the 3D-printed implant with porous structure. However, the patients with subchondral bone affected by GCTs in the proximal tibia is extremely rare. Moreover, recruiting a large number of patients in one institution is difficult, because there are few standard indications for 3D-printed prostheses at present. The complicated approval process and the high cost of 3D printing are some of the reasons why this modality is not widely used. Therefore, a larger multi-institutional study is needed to adequately compare this approach with other types of reconstruction. Despite these shortcomings, this case study may provide valuable direction for further studies.

Use of combination of 3D-printed porous implant and bone grafting can be a treatment option for Campanacci grade I or II GCTs of the proximal tibia with affected subchondral area after intracurettage. The appropriate shape of iliac graft, porous design setting of plate and strut, tight and stable connection between plate and strut, and use of three screws for fixation could lead to satisfactory biomechanical support with biological integration and prevent further damage to the subchondral area and joint. As we have collected only short-term follow-up results, the long-term efficacy of local controls for GCT and postoperative complications are yet to be observed.

## Abbreviations

3D: Three-dimensional
CT: Computed tomography
GCT: Giant cell tumor;
T-SMART: Tomosynthesis-Shimadzu metal artefact reduction technology
VAS: Visual Analog Scale
